# Effects of Chronic Swimming Training and Oestrogen Therapy on Coronary Vascular Reactivity and Expression of Antioxidant Enzymes in Ovariectomized Rats

**DOI:** 10.1371/journal.pone.0064806

**Published:** 2013-06-03

**Authors:** Erick R. G. Claudio, Patrick W. Endlich, Roger L. Santos, Margareth R. Moysés, Nazaré S. Bissoli, Sônia A. Gouvêa, Josiane F. Silva, Virginia S. Lemos, Glaucia R. Abreu

**Affiliations:** 1 Department of Physiological Sciences, Health Sciences Center, Federal University of Espírito Santo, Vitória-ES, Brazil; 2 Department of Physiology and Biophysics, Institute of Biological Sciences, Federal University of Minas Gerais, Belo Horizonte-MG, Brazil; Hosptial Infantil Universitario Niño Jesús, CIBEROBN, Spain

## Abstract

The aim of this study was to evaluate the effects of swimming training (SW) and oestrogen replacement therapy (ERT) on coronary vascular reactivity and the expression of antioxidant enzymes in ovariectomized rats. Animals were randomly assigned to one of five groups: sham (SH), ovariectomized (OVX), ovariectomized with E2 (OE2), ovariectomized with exercise (OSW), and ovariectomized with E2 plus exercise (OE2+SW). The SW protocol (5×/week, 60 min/day) and/or ERT were conducted for 8 weeks; the vasodilator response to bradykinin was analysed (Langendorff Method), and the expression of antioxidant enzymes (SOD-1 and 2, catalase) and eNOS and iNOS were evaluated by Western blotting. SW and ERT improved the vasodilator response to the highest dose of bradykinin (1000 ng). However, in the OSW group, this response was improved at 100, 300 and 1000 ng when compared to OVX (p<0,05). The SOD-1 expression was increased in all treated/trained groups compared to the OVX group (p<0,05), and catalase expression increased in the OSW group only. In the trained group, eNOS increased *vs*. OE2, and iNOS decreased *vs*. SHAM (p<0,05). SW may represent an alternative to ERT by improving coronary vasodilation, most likely by increasing antioxidant enzyme and eNOS expression and augmenting NO bioavailability.

## Introduction

Coronary heart disease (CHD) represents the major cause of morbidity and mortality in most developed countries. [1], [2] Oxidative stress, which is defined as an increase in the production of reactive oxygen species in relation to endogenous antioxidants can cause major cell damage. These compounds oxidise macromolecules such as carbohydrates, proteins, DNA and membrane lipids, [3] which is a key event in the pathogenesis of atherosclerosis, promoting endothelial dysfunction, the proliferation of vascular smooth muscle cells and the destabilisation of atherosclerotic plaques. [4] In vascular disease, this phenomenon contributes to endothelial dysfunction, mainly through the oxidative inactivation of nitric oxide (NO) by the superoxide (O_2_^•^) to form peroxinitrite (ONOO^−^) and by the uncoupling of endothelial nitric oxide synthase (eNOS), which produces O_2_^•^ rather than NO. [5] Additionally, ONOO^−^ is a potent inhibitor of prostacyclin synthesis, even at low concentrations, [6] and it impairs the activity of the Ca^2+^-activated potassium channel, which mediates the dilation induced by endothelium-derived hyperpolarising factor (EDHF). [7].

On average, women exhibit symptoms of CHD ten years later than do men, [2] and it is therefore believed that premenopausal women are protected from CHD compared to men of the same age. This cardioprotection is gradually lost after menopause. [8] These features were attributed mainly to oestrogen deficiency.

Experimental studies showed that oestrogens, especially 17-β oestradiol (E2), can exert many benefits on the cardiovascular system. For example, E2 has been shown to lower blood pressure in normotensive [9] and hypertensive [10] ovariectomized (OVX) rats and to prevent endothelial dysfunction by increasing the expression of antioxidant enzymes, [4], [11] thereby preventing oxidative stress. In addition, E2 restores flow-induced dilation in coronary arterioles, [11], [12] improves the lipid profile, [13] prevents the development of atherosclerotic lesions, [14], [15] decreases the production of pro-inflammatory cytokines [16], [17] and decreases the expression of AT_1_ receptor of angiotensin II, [10] which is a potent regulator of NADPH oxidase activity in vascular cells.

The results of large clinical trials studying hormone replacement therapy raised serious concerns about the use of this therapy [18], and it remains strongly debated. In addition to oestrogens, several studies are being conducted to investigate other compounds that have estrogenic effects on the cardiovascular system. [19], [20] In this context, lifestyle interventions such as the practice of regular exercise are very important for the control of the main risk factors for CHD, even following menopause.

Exercise training has been cited as a non-pharmacological tool to prevent or treat many cardiovascular diseases (CVD) and, in fact, it has been demonstrated that exercise can modulate a variety of risk factors for CVD. Exercise can reduce the severity of atherosclerosis in apolipoprotein E-deficient mice by improving the antioxidant system. [21] In addition, exercise enhances the production of nitric oxide, [22] augments coronary blood flow, [23] suppresses systemic low-grade inflammation, [24] and reduces blood pressure in hypertensive rats by decreasing angiotensin II levels [25] and increasing the concentration of plasma atrial natriuretic peptide. [26] In OVX animal studies, exercise training was able to positively modify body composition, [27] reverse arterial stiffness, reduce endothelin-1 levels and prevent a decrease in NO production after OVX. [28] In humans, exercise changes the cytokine production in CHD patients to an anti-inflammatory profile [29] and reduces serum glucose, LDL cholesterol and markers of oxidative stress in post-menopausal women. [30].

Nevertheless, little is known about the relationship between exercise and coronary vascular reactivity in female OVX rats. We hypothesise that exercise training could prevent and/or improve the impaired response in vasodilation promoted by bradykinin, observed with oestrogen deficiency in the coronary arterial bed to the same extent as observed in experimental studies with oestrogen replacement therapy (ERT). We analysed the expression of antioxidant enzymes to verify one of the possible mechanisms associated with exercise-mediated improvements in vasodilator response.

## Methods

### Animals

Female normotensive Wistar rats with 8 weeks of age, weighing between 230–240 g were given by the university facility. All procedures were approved by the Institutional Ethical Committee for Animal Care and Use of the Federal University of Espírito Santo under protocol number 024/2011. Experiments were conducted in accordance with the Guide for the Care and Use of Laboratory Animals published by the US National Institutes of Health (NIH Publication, revised 1996) and efforts were made to minimize the animals suffering. The animals were keeping in collective cages with free access to water and standard rat chow (Purina Labina®, SP, Brazil), under controlled temperature (22–24°C), humidity (40–60%) and light-dark cycle (12–12 h). At the time of ovariectomy the animals were randomly divided in five groups as following: Sham (SH), n = 15; ovariectomized (OVX), n = 20; ovariectomized with E2 replacement (OE2), n = 20; ovariectomized and swimming training (OSW), n = 24 and ovariectomized with E2 replacement plus swimming training (OE2+SW), n = 20.

### Ovariectomy

Ovariectomy was performed under general anesthesia with ketamine (80 mg/kg) and xylazine (12 mg/kg) *i.p*. Bilateral dorsolateral incision was made through skin and the underlying muscle was dissected to locate the ovary and fallopian tube. The tube was ligated with a suture line and the ovary was removed. The muscle and skin were then sutured with an absorbable suture. After the surgery animals received an injection of antibiotic (2,5% enrofloxacin, 0,1 mL, *i.m*). In sham animals it was made a fictitious surgery. All the animals were submitted to surgery in the same period and started the swimming training and ERT after seven days of recovery. Early initiation of ERT and training protocol after ovariectomy was made to prevent the increase in oxidative stress and in pro-inflammatory cytokines levels, as reported in rats with delayed start of ERT. [31].

### Oestrogen Replacement Therapy

Oestrogen replacement therapy was performed by subcutaneous injections (0,1 mL), containing 5 µg of 17β-oestradiol 3-benzoate (Sigma, St Louis, MO) diluted in corn oil, 3 times per week, as previously described. [32] Animals that did not receive ERT had the same volume injected containing corn oil only. Effectiveness of ovariectomy and ERT was assessed by plasma 17β-oestradiol concentration and uterine wet weight.

### Swimming Training

The swimming training protocol was performed in an apparatus adapted for rats containing warm water (30–32°C) and the depth was kept in 60 cm. The training protocol was conducted in the same period of the day (16∶00–18∶00 pm) in all of the training sessions. In the first week the animals were submitted to an adaptation period consisting in twenty minutes of continuous swimming training in the first day, which was increased daily by ten minutes until they reached sixty minutes on the fifth day. From the second week, the exercise duration was kept constant (60 min/day, 5 days/wk) with two days of rest, until the end of training period that lasted eight weeks. Animals rested for 48 h (to analyze the effects of chronic exercise) before the sacrifice for all procedures. [33].

### Isolation of Coronary Arteries

The animals were sacrificed by decapitation. The thorax cavity was open, the heart removed and placed in cold Krebs-Henseleit solution buffer (in mmol/L): 115 NaCl, 25 NaHCO_3_, 4,7 KCl, 1,2 MgSO_4_.7H_2_O, 2,5 CaCl_2_, 1,2 KH_2_PO_4_, 5,5 glucose and 0,01 Na_2_EDTA) at pH 7.4 during the dissection procedure. The left anterior descending branch of left coronary artery and the septal branch were isolated in a dissection microscope (D.F. Vasconcelos M900, São Paulo, Brazil) free of surround ventricular muscle tissue and snap frozen in liquid nitrogen. Afterwards, the samples were stocked at −80°C until their use.

### Western Blotting

The coronary arteries were pooled with frozen tissue of three animals samples (n = 1). The samples were homogenized in lysis buffer, containing (in mmol/l) 150 NaCl, 50 Tris-HCl, 5 EDTA.2Na, 1 MgCl2 plus protease inhibitor (Sigma Fast; Sigma, USA). The protein concentration was determined by Lowry method, [34] and bovine serum albumin (BSA) was used as standard. Equal amounts of protein were denatured and separated by electrophoresis SDS-PAGE/10% and transferred onto a nitrocellulose membrane (Millipore). The membranes were blocked with 5% BSA at room temperature in TBS buffer plus Tween 20 (0,1%) before incubation with polyclonal anti-goat for superoxide dismutase 1 - SOD-1 (1∶2500-Sigma), and SOD-2 (1∶2000-Sigma), monoclonal anti-mouse for Catalase (1∶2000-Sigma), monoclonal anti-mouse for eNOS (1∶1500-BD) and iNOS (1∶1500-BD) and polyclonal anti-mouse for β-actina (1∶1500-Santa Cruz Biotechnology). Immunoreactive bands were detected with chemiluminescence reaction using peroxidase substrate (Luminata HRP Substrate-Millipore) and then exposed to X-ray film. The densitometric analysis was made by ImageJ software (National Institute of Health).

### Isolated Heart Preparation (Modified Langendorff Method)

To assess coronary perfusion pressure (CPP) and the endothelium-dependent vasodilation, the animals were anesthetized with chloral hydrate (40 mg/kg, *i.p*). The rats were killed, the heart excised and immediately perfused at a constant flow. The studies on the coronary vascular bed were performed on whole hearts using a modified Langendorff preparation for perfused isolated hearts as previously described. [35] Briefly, using a Langendorff apparatus (Hugo Sachs Electronics, March-Hugstetten, Germany), the isolated hearts were perfused with modified Krebs solution containing (in mM): NaCl, 120; CaCl_2_.2H_2_O, 1,25; KCl, 5,4; MgSO_4_.7H_2_O, 2,5; NaH_2_PO_4_.H_2_O, 2,0; NaHCO_3_, 27,0; Na_2_SO_4_, 1,2; EDTA, 0,03 and glucose 5,5 equilibrated with a 95% oxygen and 5% carbon dioxide mixture at a controlled pressure of 100 mmHg to give a pH of 7.4, perfused at a rate of 10 ml/min with a peristaltic pump (MS-Reglo 4 channels, Hugo Sachs Electronics), and kept at 37°C. A fluid-filled balloon was introduced into the left ventricle through a steel cannula with a latex balloon and connected to a TPS-2 Statham transducer (Incor, São Paulo, SP, Brazil) to measure the isovolumetric force. The balloon was pressurized with a spindle syringe until it reached a preload of 10 mmHg. CPP was monitored with a TPS-2 Statham transducer connected to a sidearm of the aortic perfusion catheter. After the stabilization period (40 min), baseline CPP was measured. The endothelium-dependent vasodilation was analyzed in coronary arterial bed, randomly, through in bolus administration (0,1 mL) of bradykinin (Sigma, St. Louis, MO) in the following concentrations (0,1; 1; 10; 100; 300 e 1000 ng).

### Plasma 17β-oestradiol Concentrations

After decapitation, blood samples were collected in sterile tubes containing EDTA/K3, centrifuged at 3.000 *g* during 15 min at 4°C (Fanem, São Paulo, Brazil) and stored at −80°C until use. Plasma 17β-oestradiol concentrations were analyzed by electrochemiluminescence immunoassay method (Elecsys 2010, Roche, Basel, Switzerland), with available kits (Estradiol II, Roche, Mannheim, Germany).

### Statistical Analysis

Data are reported as mean ± SEM. Data for organ weights, enzyme expression and baseline CPP were analyzed by one-way analysis of variance (ANOVA) considering the treatment (training and/or estrogen) as the main factor. The endothelium-dependent vasodilation was tested by the two-way ANOVA, where the treatment and the concentrations of BK employed were the factors. In both cases, the differences among groups were tested by the Fisher’s *post-hoc* test for multiple comparisons. Statistical significance was set at p<0,05.

## Results

### Surgery and Training Efficacy

Plasma 17β-oestradiol concentration, the uterus weight (UW) and the ratio of UW to body weight (BW) were used to determine the estrogenic status. As expected, there was a significant decrease in all of these parameters in OVX animals (p<0,05), and this decrease was prevented by E2 treatment (Table 1). Table 1 also shows that the heart weight (HW) and HW to BW ratio were significantly increased in the female rats in the trained groups (p<0,05). This hypertrophic response is an expected physiological adaptation to exercise training, therefore proving its efficiency.

**Table 1:** Effects of ovariectomy, swimming training and estrogen therapy on weight and body composition, estrogenic status and heart weight.

	SH	OVX	OE2	OE2+SW	OSW
N	15	20	20	20	24
**BW initial (g)**	227±5	236±4	239±6	238±6	234±3
**BW final (g)**	277±10a,b	328±12	279±9a,b	285±5^b^	309±8
**BW range (%)**	24.42±3.46a,b	38.61±2.78	18.39±2.96a,b	17.18±2.90a,b	34.37±3.24
**RF (g)**	4.31±0.88	6.05±0.82	4.10±0.34	3.30±0.50^b^	3.99±0.91^b^
**PF (g)**	6.17±0.7	5.96±0.95	5.39±0.74	4.25±0.35	3.79±0.39b,e
**RF+PF (g)**	10.48±1.46	12.01±1.36	9.48±3.24	7.55±2.14^b^	7.79±1.20^b^
**UW (mg)**	527±65	145±42c,d,e	584±145	669±50	161±34c,d,e
**UW/BW (mg/g)**	1.99±0.24	0.45±0.12c,d,e	2.14±0.14	2.41±0.77	0.52±0.11c,d,e
**E2 (pg/mL)**	48±7.68	19.78±1.64 c,d,e	49.25±6.71	60.67±7.03	29.64±4.43c,d,e
**HW (mg)**	844±33	857±28	866±49	1033±33b,d,e	1091±32b,d,e
**HW/BW (mg/g)**	3,06±0,1^b^	2,67±0,09	3,10±0,15^b^	3,64±0,12b,d,e	3,57±0,15b,d,e

BW, body weight; RF, retroperitoneal fat; PF, parametrial fat; UW, uterine weight; UW/BW uterine and body weight ratio; HW, heart weight and HW/BW, heart and body weight ratio.

Data are expressed as mean ± SEM.

a p<0,05 vs OSW;

b p<0,05 vs OVX;

c p<0,05 vs OE2+SW;

d p<0,05 vs OE2.

e p<0,05 vs SH.

### Body Weight and Adiposity

Weight and adiposity, as analysed in Table 1, showed a smaller percentage of increase in BW (Δ%BW) in OVX rats that were treated with E2 (OE2 and OE2+SW) (p<0,05) compared to sedentary OVX and OSW rats. However, analyses of the parametrial and retroperitoneal fat deposits and their sums show that only SW prevents the excessive fat accumulation that occurs with OVX; the fat weights in the two trained groups were significantly smaller than that in the OVX group (p<0,05), demonstrating the efficiency of SW in reducing adiposity.

### Baseline CPP and Vasodilator Response to Bradykinin

**Figure 1:**
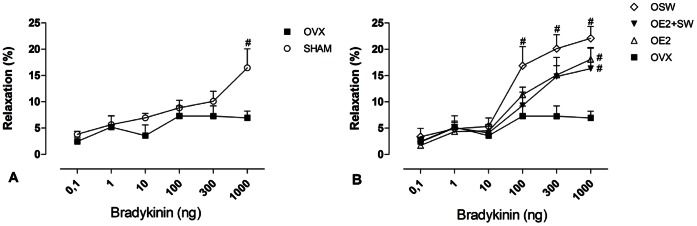
Vasodilator response to growing concentrations of bradykinin (0,1–1000 ng). (A) Sham group had higher vasodilator response compared to OVX at 1000 ng (#p<0,05). (B) All trained/treated groups had a significant increased vasodilator response at the highest concentration (1000 ng) compared to OVX group (#p<0,05). In the OSW group, the vasodilator response was significant in the three highest concentration when compared to OVX group (#p<0,05). Data are expressed as mean ± SEM; n = 6–9 animals per group.

The baseline CPP was not different among groups (SHAM: 87.11±2.88; OVX: 79.74±3.38; OE2∶83.47±4.66; OE2+SW: 76.15±3.96; OSW: 82.52±5.43 mmHg). However, OVX decreased the dilation induced by bradykinin when compared to Sham operated animals (p<0,05) at 1000 ng (two-way ANOVA, df = 4; F = 3,604; p = 0,015) (Figure 1A). All treatments (E2, SW and E2 plus SW) were able to prevent this decrease caused by OVX (Figure 1B). The OSW group exhibited more pronounced vasodilatory responses than did the OVX group, and the differences in responses were significant (p<0,05) at the three highest concentrations of bradykinin (two-way ANOVA to 100 ng, df = 4; F = 1,591; p = 0,199 and 300 ng, df = 4; F = 2,630; p = 0,055). In contrast, for each of the other groups (OE2 and OE2+SW), the difference in the vasodilatory response compared with OVX was significant (p<0,05) only at the maximum concentration (1000 ng).

### Antioxidant Enzymes Expression

**Figure 2:**
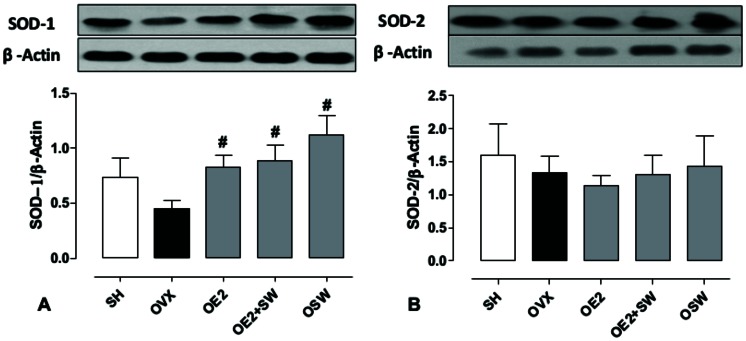
Expression of superoxide dismutase isoforms in coronary arteries. (A) Cytosolic isoform (SOD-1), showing significant difference in all trained/treated groups compared to OVX group (#p<0,05). (B) Mitochondrial isoform (SOD-2), demonstrating no statistical differences among groups. Data are expressed as mean ± SEM; n = 3–5 animals per group.

**Figure 3:**
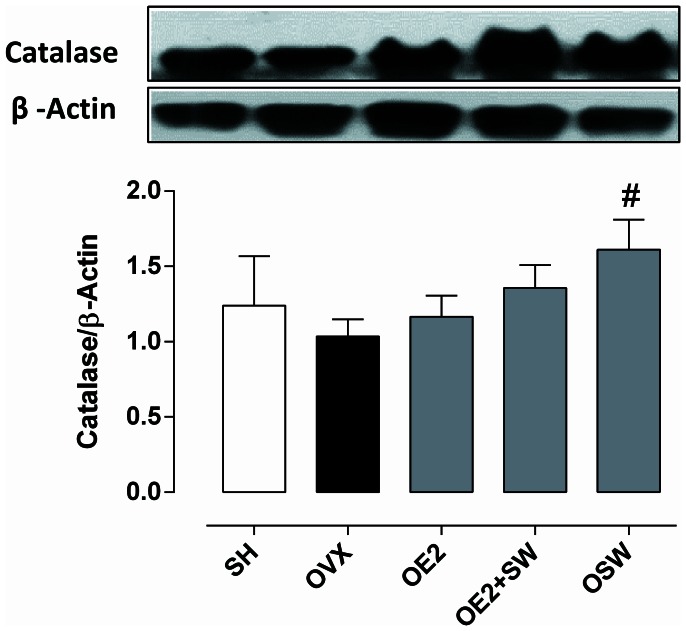
Coronary expression of the antioxidant enzyme catalase. There were significant increased expression only in the trained group (OSW) when compared to OVX group (#p<0,05). Data are expressed as mean ± SEM; n = 3–5 animals per group.

**Figure 4:**
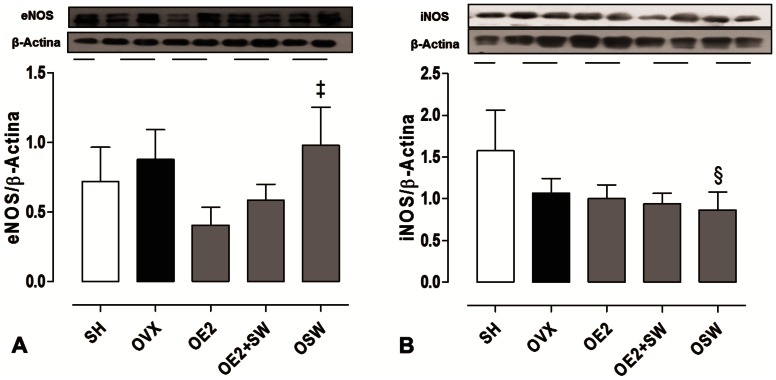
Expression of nitric oxide synthase isoforms in coronary arteries. (A) The endothelial isoform of nitric oxide synthase (eNOS) showing increased expression in OSW group compared to OE2 group (‡p<0,05) and (B) Inducible isoform of nitric oxide synthase (iNOS). In OSW group its expression was significantly decreased in relation to SH group (§p<0,05). Data are expressed as mean ± SEM; n = 3–5 animals per group.

The expression of antioxidant enzymes on coronary arteries was verified to analyse the possible role of antioxidant status in endothelium-dependent vasodilation. Cu/Zn-superoxide dismutase (SOD-1) expression (Figure 2A), which catalyses the dismutation of superoxide anion (O_2_^•^) to hydrogen peroxide (H_2_O_2_), was significantly increased in all of the treated groups (OE2, OE2+SW and OSW) groups compared to OVX (p<0,05), suggesting that its expression in coronary arteries can be regulated by both exercise and E2. However, the expression of its mitochondrial isoform (SOD-2) was not different among the groups studied (Figure 2B). The expression of catalase (Figure 3), which decomposes H_2_O_2_ into water and oxygen, was significantly increased only in the OSW group compared to the OVX group (p<0,05). Therefore, it seems that its vascular expression is regulated mainly by exercise-induced oxidative stress. The expression of eNOS did not increase with E2 treatment (Figure 4A), and only the OSW group exhibited a significant increase in eNOS expression compared to the OE2 group (p<0,05). iNOS isoform expression decreased in the OSW group only (Figure 4B) compared to the SHAM (p<0,05).

## Discussion

The aims of the present study were to analyse the effects of chronic SW and ERT on the bradykinin-mediated vasodilation of the coronary vascular bed in female OVX rats and to verify the possible role of antioxidant enzyme expression on these responses.

Currently, other alternatives to protect the cardiovascular system and prevent CHD becomes of great importance. Although the studies performed experimentally showed beneficial effects, [9], [11], [12] it was demonstrated clinically that ERT do not provide protection against myocardial infarction and CHD [18] and may even increase the risk for breast cancer development. [36].

Accordingly, the main findings of this study was that chronic SW and ERT can prevent the decrease in the vasodilator response promoted to bradykinin as well as enhance the protein expression of some antioxidant enzymes in the coronary arteries of OVX rats. This may be one of the main mechanisms related to the benefits of exercise and ERT on coronary vascular function and in the prevention of CHD.

In addition to this vascular mechanism, adiposity is another important factor that can enhance the risk of CHD in the postmenopausal period, as verified in this work. During the training/treatment protocol, the OVX group that did not receive ERT exhibited a significant increase in BW. In a previous study, [37] which analysed the possible causes involved in this phenomenon with E2 deficiency, the authors observed that after OVX, the animals exhibit hyperphagic behaviour and reduced locomotor activity, and therefore, they are more prone to accumulating fat because of changes in behaviour. In addition, lipoprotein metabolism is altered in postmenopausal women, in whom the rate of lipolysis is decreased and the activity of lipoprotein lipase in adipose tissue is augmented. [38].

ERT prevents weight gain in OVX rats, but SW does not. However, SW was able to change the body composition, as demonstrated by the fat deposit weights. Similar results with exercise training were found in OVX rats by Shinoda et al. [27] and in OVX Wistar rats with diet-induced obesity by Zoth et al. [39] although these authors did not evaluate precisely the same fat deposits investigated in our study. Similar results are also observed in humans; women undergo an increase in BW during the menopausal transition period, in addition to changes in body composition. [40], [41] However, these effects can be counteracted by both ERT [40], [42] and exercise training. [43], [44].

Nevertheless, consistent with our hypothesis, the vasodilation in the coronary bed in response to the highest concentration of bradykinin was significantly higher in all treated/trained groups compared to OVX rats. However, this response was more pronounced in the trained group (OSW), in which the vasodilation was significantly higher than that of OVX at the three highest concentrations. The increased expression of the antioxidant enzymes SOD-1 and catalase in the OSW group may be the major factor associated with this result.

Indeed, the protein expression of SOD-1 increased in response to SW and ERT. This fact is particularly important because SOD-1 activity accounts for approximately 50–80% of all SOD isoform activity in the vascular wall. [45], [46] In studies of ERT, it was verified that the expression of this enzyme in coronary arterioles decreases with OVX, which was prevented by E2 treatment. [11] Strehlow et al. [4] reported that, in cultured rat aorta vascular smooth muscle cells and in human monocytes, incubation with E2 enhances the mRNA content and protein expression of SOD-2 but has no effect on SOD-1, contrary to our observations in coronary arteries. Additionally, other study [47] showed no difference on SOD-1 expression in the aorta of SHR animals that were submitted to ovariectomy, but increased when treated with conjugated equine estrogen. Taken together, these results demonstrated that the role of estrogen on vascular expression of this enzyme remains inconclusive, and further studies are needed. In contrast, animal studies have shown that the vascular expression of SOD-1 is augmented upon exercise training in many species, including in the coronary arteries of pigs, [48], [49] in the aorta and in mesenteric bed of rats with diet-induced obesity [50] and in the aorta of diabetic mice. [51] In all of these cases, improvements in endothelium-mediated dilation have been reported, suggesting that this enzyme is in fact of great importance for the maintenance of endothelial function and that its expression in response to ERT seems to be dependent on the vessel analysed.

The mechanism that seems to regulate this response in exercised rats is the increase in endothelial mechanical stress, as demonstrated by Inoue et al. [52] who showed that shear stress is indeed capable of modulating SOD-1 expression in human aorta endothelial cells but not in smooth muscle.

Although the treatments are capable to increase the expression of SOD-1 and the vasodilatory response, they are not cumulative. Maybe, because of the treatments made in an isolated manner were enough to maintain ROS homeostasis, the addition of both does not require additional effects.

It is believed that the increase of SOD-1 is accompanied by augmented H_2_O_2_ production. Peroxidases like catalase have an essential role in the maintenance of H_2_O_2_ homeostasis in cells, converting this molecule into water and oxygen. [53] As shown in Figure 3, the expression of catalase increased only in the trained group compared to the OVX group. Consistent with our findings, Kang et al. [11] reported no changes in the coronary expression of catalase in response to ERT, and Xu et al. [54] reported enhanced catalase expression and activity in cardiac muscle after exercise training in rats submitted to myocardial infarction.

Recently, the cellular functions of H_2_O_2_ have been described in more detail. This molecule can modulate the coronary vasodilation induced by cardiac metabolism in rats [55] and also acts as an endothelium-derived hyperpolarising factor in the vascular beds of many species. [46], [56] Furthermore, H_2_O_2_ has already been described as a signalling molecule, a regulator of gene expression, and a selective activator of transcription factors [45] such as nuclear factor kappa B (NFkB). NFkB regulates the gene transcription of pro-inflammatory molecules, and its chronic stimulation through the enhancement of oxidative stress can therefore promote the development of atherosclerosis. [57] Studies in ApoE KO mice showed a reduction in the development of atherosclerosis in animals that overexpress catalase or catalase and SOD-1 but not in those overexpressing only SOD-1, suggesting that H_2_O_2_ has a greater atherogenic potential than O_2_^−^, most likely through its signalling function on NFkB. [58], [59] As we observed that catalase expression was increased in the SW group, this may be the mechanism by which iNOS expression was decreased, providing further evidence for the anti-inflammatory effects of exercise.

Another factor that may justify the improvements in vasodilation in conjunction with antioxidant enzyme expression was reported by Suvorava et al. [60] These authors demonstrated that the increase in H_2_O_2_ production induced by exercise decreases the number of circulating endothelial progenitor cells in mice. However, in animals that overexpress catalase, the number of these cells is increased after exercise, suggesting that this enzyme can assist in endothelial repair.

It has also been reported that the H_2_O_2_ production induced by exercise exerts a positive regulatory role on eNOS expression [61] through a mechanism that is dependent on Ca^2+^/Calmodulin-dependent protein kinase-II and JNK-2. [62] However, this does not seem to be the mechanism involved in the induction of the expression of this enzyme by exercise, at least in the present study, because catalase expression is increased in the exercising group, probably reducing the H_2_O_2_ concentration. In addition to the mechanisms cited above, the laminar shear stress is another potent stimulator of the expression and mRNA stabilisation of catalase [63] and increases its activity by multiple pathways. [64], [65] As exercise admittedly increases the tension exerted on the vascular wall (and, consequently, the release of NO), the elevation of this vasodilatory factor is most likely the key factor for the increase in vasodilation in the SW group. Other studies have shown that the vascular expression of eNOS is, in fact, enhanced with exercise training, [66], [67] leading to an improvement in endothelium-dependent vasodilation, even with the chronic inhibition of this enzyme. [67].

Thus, our results support the hypothesis that the chronic SW can improve the vasodilator response promoted by bradykinin to the same extent as ERT. These results suggest that the main alterations observed in these responses seem to occur at endothelial level, once that the vasodilatory response promoted by bradykinin is endothelium-dependent. One of the mechanisms that may be associated with these effects is the increased expression of antioxidant enzymes, which prevents the vascular oxidative stress induced by E2 deficiency and the consequent cytotoxic effects.

A potential limitation of this study is that ovariectomy was made at eight-week old rats. At this age the animals are considered young adult and do not reached the sexual maturity. However, the present data clearly support the negative vascular effects of oestrogen deficiency even with the age limitation.

### Conclusions

These data support the theory that the SW and ERT may play an important role in coronary vascular reactivity and in the expression of antioxidant enzymes, which may be one of the reasons why exercise and ERT reduce the risk of coronary heart disease in postmenopausal women. Furthermore, chronic exercise training may be a feasible alternative to ERT for preventing CHD in postmenopausal women.
